# The water-related traits of flowers are more conservative than those of leaves for epiphytic and terrestrial species in *Cymbidium*, Orchidaceae

**DOI:** 10.1093/aobpla/plaf033

**Published:** 2025-06-24

**Authors:** Feng-Ping Zhang, Cui-Ying Chen, Jia-Lin Huang, Hong Hu, Shi-Bao Zhang

**Affiliations:** Yunnan Key Laboratory of Dai and Yi Medicines, College of Ethnic Medicine, Yunnan University of Chinese Medicine, Kunming 650500, Yunnan, China; Yunnan Key Laboratory of Dai and Yi Medicines, College of Ethnic Medicine, Yunnan University of Chinese Medicine, Kunming 650500, Yunnan, China; Yuxi Normal University, Yuxi, China; Key Laboratory of Economic Plants and Biotechnology, Yunnan Key Laboratory for Wild Plant Resources, Kunming Institute of Botany, Chinese Academy of Sciences, Kunming 650201, Yunnan, China; Key Laboratory of Economic Plants and Biotechnology, Yunnan Key Laboratory for Wild Plant Resources, Kunming Institute of Botany, Chinese Academy of Sciences, Kunming 650201, Yunnan, China; Form & Function

**Keywords:** morphological and physiological traits, adaptation strategy, floral traits, life form, epiphytic orchids

## Abstract

Epiphytes occupy arboreal niches in forest ecosystems, which are particularly vulnerable to drought stress due to the absence of a buffered substrate for water retention in epiphytic habitats. Characterizing the differences and relationships among plant morphological and physiological traits is critical for elucidating different adaptive strategies. However, it is still unclear whether there are differences in floral and leaf morphological and physiological traits between epiphytic and terrestrial plants, and whether there is a correlation between flower and leaf traits in epiphytes. Here, we measured 13 floral traits and 8 leaf traits from 7 terrestrial and 12 epiphytic *Cymbidium* species. We found that, compared with these terrestrial *Cymbidium* species, epiphytic species had a higher leaf mass per unit area, greater leaf thickness, a longer time required to dry saturated leaves to 70% relative water content, and greater epidermal thickness. However, no significant differences in floral traits were found between the epiphytic and the terrestrial species, which suggest that the water-related traits of flowers in *Cymbidium* are not influenced by the plant's life forms. Moreover, there were no strong associations between floral and leaf morphological and physiological traits floral traits, implying that they may be developmentally modular. These findings provide novel insights into the decoupled evolution of vegetative and reproductive traits in response to environmental pressures. By shedding light on this pattern, our study advances the understanding of plant adaptation strategies in heterogeneous habitats within the genus *Cymbidium*, providing a more comprehensive view of how plants evolve to flourish in diverse ecological conditions.

## Teaser

Our results showed epiphytic Cymbidium species had thicker, heavier leaves with tougher epidermis and slower water loss, but their flowers showed no differences from terrestrial species. Flower and leaf traits were also unrelated, suggesting they evolve independently. These findings provide insights into the decoupled evolution of vegetative and reproductive traits in response to environmental pressures.

## Introduction

Epiphytes are important contributors to the biodiversity of tropical and subtropical mountain forest ecosystems and play a crucial role in maintaining water, carbon, and nutrient balance of ecosystems (Liu et al. 2006, Zotz and Bader 2009). Nearly 70% of the family Orchidaceae species are epiphytic to the canopy, which is often subject to drought compared with terrestrial species owing to the limited substrate availability in epiphytic habitats (Benzing 1990, Silvera et al. 2009). Therefore, water shortage is one of the most limiting factors of the survival, growth, and reproduction of epiphytes (Benzing 1990, Zotz and Hietz 2001, Laube and Zotz 2003).

Epiphytic plants have evolved diverse morphological and physiological traits to cope with water stress. Epiphytes mainly utilize water from canopy mats and from occult precipitation (Feild and Dawson 1998). The water storage capacity of plant organs may be the main strategy for epiphytic species to cope with water deficit (Munné-Bosch et al. 1999, Zotz and Bader 2009, Limm and Dawson 2010, Reyes-Garía et al. 2012). This greater storage capacity leads to the maintenance of more stable water potentials in plants during periods of drought (Goldstein et al. 1998, Stratton et al. 2000, Pivovaroff et al. 2014). Storage capacity is associated with leaf morphological traits (Ogburn and Edwards 2010, 2013, Guan et al. 2011, Zhang et al. 2012, 2015, Pivovaroff et al. 2014). For example, thicker leaves can reduce the rate of leaf water conductance, while thicker leaf cuticles can slow down the rate of water loss caused by epidermal transpiration (Kerstiens 1996, Riederer and Schreiber 2001). In addition, a higher leaf vein density (LVD) can improve leaf water transport efficiency (Sack and Frole 2006).

Epiphytic species growing in canopy habitats experience more dramatic fluctuations in vapour pressure deficits than those in terrestrial habitats (Watkins and Cardelús 2012). Water shortage is one of the most limiting factors for the establishment and growth of epiphytic species (Benzing 1990, Zotz and Hietz 2001, Laube and Zotz 2003). The epiphytic *Cymbidium tracyanum* had a higher ratio of velamen thickness to root thickness and a longer time required for drying of saturated leaves to 70% relative water content (RWC; *T*_leaf 70_) than those of *Cymbidium sinense*. This showed that *C*. *tracyanum* has a greater capacity to conserve water and mitigate the effects of drought stress (Chaves et al. 2002, Li et al. 2018). Furthermore, the water potential at the turgor loss point (Ψ_TLP_) of *C*. *tracyanum* was lower than that of *C*. *sinense*, indicating that the epiphytic orchid, *C*. *tracyanum*, has a greater capacity for drought tolerance (Li et al. 2018). The increase in phytohormone concentration and antioxidant activity of *C*. *sinense* helped this terrestrial species to survive under water stress (Li et al. 2018). However, previous studies have mainly focused on the differences in leaf water relations (Rada and Jaimez 1992, Watkins and Cardelús 2012, Zhang et al. 2015), without considering other important tissues, such as flowers, between epiphytic and terrestrial species.

Flowers are one of the most important reproductive organs in plants (Rosas-Guerrero et al. 2014), which differ significantly from leaves in function (pollinator attraction) and development. Although flowers contribute little to carbon assimilation, their maintenance still requires a considerable amount of water supply (Roddy and Dawson 2012, Teixido and Valladares 2014, Roddy et al. 2016, 2018). Monocots and eudicots indicated relatively conservative flower water use strategies with low water flux and low carbon investment, whereas flowers of basal angiosperms evolved an efficient water transport system, characterized by the high density of flower veins and stomata (Ke et al. 2024). Under drought conditions, prolonged water stress shortens flower lifespan (Arathi et al. 2002), and flowers are more susceptible to water stress than leaves, and drought is more likely to disrupt flower water transport functions (Zhang and Brodribb 2017). A previous study suggests that the hydraulic structural characteristics and water use strategies of flowers and leaves are different (Roddy et al. 2013). For example, the independent evolution of vein densities in leaves and petals implies that these organs may operate as developmentally modular units, each with its own evolutionary trajectory and functional specialization (Roddy et al. 2013).

However, despite their important role, floral morphological and physiological traits have received less attention than leaves, and we know little about how floral morphological and physiological traits respond to drought stress in epiphytic species. Unlike leaves, most petals are not major sites of carbon assimilation and thus contribute relatively little to photosynthesis. However, flowers are exposed to the same evaporative environment similar to that of leaves, which means they require a continuous water supply throughout their lifespan. As a result, there are trade-offs between the resources invested in flowers and those invested in leaves (Bazzaz et al. 1987, Reekie and Bazzaz 1987). Water for flowers has to be supplied by roots and stems, and this process may potentially come at the cost of vegetative functions (Nobel 1977, Galen et al. 1999, Lambrecht and Dawson 2007). Therefore, understanding the morphological and water physiological traits of flowers could potentially uncover different perspectives on the evolution of flowers (Roddy et al. 2013).

Members of the genus *Cymbidium* (Orchidaceae) can be epiphytic or terrestrial, which are of significant ornamental, commercial, and conservation value (Zhang et al. 2001, Zhou et al. 2020). Some species of *Cymbidium* are endangered species (Luo et al. 2003, Liu et al. 2009). Therefore, investigating adaptive rules by which *Cymbidium* species respond to environmental stress would be beneficial for their conservation and the cultivation of new varieties in *Cymbidium*, in order to understand the adaptive strategies of orchid plants. *Cymbidium* species may develop different mechanisms that adapt to their growing environments (Zhang et al. 2015, Li et al. 2018). The terrestrial orchid, *C*. *sinens*e, seemingly adopts a ‘remedy strategy’ in response to drought stress. In contrast, the epiphytic orchid, *C*. *tracyanum,* employs a ‘precaution strategy’ against water stress (Li et al. 2018).

Compared with terrestrial species, epiphytic species had higher values of leaf mass per unit area (LMA), leaf thickness (LT), and time required for drying of saturated leaves to 70% RWC, the ratio of velamen thickness to root thickness, and larger water storage cells and higher water content, indicating greater drought resistance and water storage capacity of their leaves, roots, and pseudobulbs (Zhang et al. 2015, Li et al. 2018, Li and Zhang 2019). Previous studies mainly focused on the morphological and physiological traits of the vegetative organs, exploring their adaptation strategies to epiphytic habits in water-limited environments. Like leaves, flowers are also confronted with the same environmental and resource constraints, requiring a continuous water supply throughout their developmental stages. Consequently, it is expected that epiphytic orchids might exhibit floral trait values indicative of greater drought tolerance and increased water storage capacity compared with terrestrial orchids.

Here, we used a common garden experiment to assess 21 flower and leaf morphological and physiological traits in closely related species, including 7 terrestrial and 12 epiphytic *Cymbidium* species (Supplementary Fig. S1). The aim of this study was to ascertain the differences in leaf and flower characteristics between the epiphytic and the terrestrial *Cymbidium* species and to comprehend the adaptive mechanisms used by these two life-form species to occupy their habitats. Specifically, we were interested in the following scientific questions: (i) Do the 19 *Cymbidium* species from the two life forms differ in floral and leaf morphological and physiological traits? (ii) Are their coordinated relationships between morphological and physiological traits in flowers and leaves?

## Materials and methods

### Plant materials

Nineteen *Cymbidium* species, including 7 terrestrial and 12 epiphytic species, were selected. The life forms, elevation, and habitats are shown in Supplementary Table S1. These species were grown in a greenhouse under the same growth conditions (the relative humidity: 60%–70%, day temperatures: 20–25°C, night temperatures: 10–15°C) at the Kunming Institute of Botany, Chinese Academy of Sciences. They were grown in plastic plots with a mixture of identical substrates (70% bark, 20% moss, and 10% humus), which are watered 1–2 times per week, and pouring a permeable each time, and their cultivation substrates were check daily, thus to ensure they are not subjected to water stress.

### Measurements of flower and leaf physiological traits

To measure the rate of water loss of flower and leaf, six fresh flowers (*n* = 6) and six healthy leaves (*n* = 6) from six different plants of each species were collected. The collected flowers and leaves were placed in distilled water after recutting pedicels and petioles to completely rehydrate, the saturated fresh weight (SFW) was measured, and the flowers and leaves were then placed in an incubator temperature: 24°C, relative humidity: 60%. Fresh weights (FWs) were measured periodically using a digital balance until the weight remained constant. The floral area (FA) of all organs in a flower, i.e. petal, sepal, and lip, as well as the leaf area (LA), was measured using a Li-Cor 3000A area meter (Li-Cor, Inc., USA). Finally, the flowers and leaves were dried under 70°C for 48 h to determine dry weight (DW). The RWC was calculated as follows: (FW − DW)/(SFW − DW) × 100 (Zhang et al. 2015). The time needed to dry a saturated flower/leaf to 70% RWC (*T*_flower70_ and *T*_leaf70_) was determined by regressing RWC against the time interval from leaf excision to each measurement of FW (Hao et al. 2010, Zhang et al. 2015). Floral dry mass per unit area (FMA, g m^−2^) was calculated as DW/FA. LMA (g m^−2^) was calculated as DW/LA.

To monitor floral longevity (FL), 10–29 floral buds from at least three individuals of each species with racemes each species were marked, which were checked twice per day. FL was regarded as the length of time from opening to closing (Zhang et al. 2017, Roddy et al. 2021, Paiva and Roddy 2024). The value of FL for each species was taken by averaging the time each flower remained open (Paiva and Roddy 2024).

### Measurements of flower and leaf morphological traits

Six flowers (*n* = 6) from at least three different plants of each species are preserved in FAA (a formalin acetic acid–alcohol solution: 37% formaldehyde, glacial acetic acid, 95% ethanol, and deionized water in a 10:5:50:35). The transverse cross sections of the petal, sepal, and leaf were examined and photographed at 10× magnification using a Leica DM2500 microscope (Leica, Wetzlar, Germany). The values for upper epidermal thickness of petals, sepal, and leaf (PUET, SUET, and LUET), lower epidermal thickness of petals, sepal, and leaf (PLET, SLET, and LLET), mesophyll thickness of petals, sepal, and leaf (PMT, SMT, and LMT), and petal thickness (PT), sepal thickness (ST), and LT were determined using ImageJ 1.47v.

Six petals (*n* = 6) and six sepals (*n* = 6) were sampled and scanned at 2400 dpi using a scanner. Vein densities of petals and sepals were measured using the ImageJ software as the total length of vascular tissue per mm^2^ of their surface area. The collected leaves (*n* = 6) were soaked in a 5% NaOH aqueous solution to remove mesophyll tissue for measuring the LVD. Samples of leaves were photographed at 10× magnification with a digital camera mounted on a Leica DM2500 microscope (Leica Microsystems Vertrieb GmbH; Zhang et al. 2015). Vein lengths were determined from digital images via the ImageJ software and the values. Values for vein density were expressed as vein length per unit area. Leaf stomatal density (LSD) was photographed using a Leica DM2500 microscope. Stomata were observed in 30 randomly selected fields of view, and LSD was calculated as the number of stomata per unit area (Zhang et al. 2015).

### Statistical analysis

Differences in flower and leaf morphological and physiological traits between epiphytic and terrestrial *Cymbidium* species were examined using the independent sample *t*-test in the ‘stat’ package in R v.4.3.0 (R Core Team 2020). The coefficient of variation (CV) was (%) = [(standard deviation/mean) × 100%). Relationships among traits were tested by using Pearson correlation.

The phylogenetic tree of *Cymbidium* species was constructed based on the complete chloroplast genome sequences from the NCBI database. These sequences were aligned using MAFFT v7.450 (Katoh and Standley 2013), and a phylogenetic tree was constructed using the fasttree software (version 2.1.11 SSE3; http://www.microbesonline.org/fasttree/) with the Maximum likelihood method using the GTR + GAMMA model with 1000 ultrafast bootstraps replicates. In order to account for phylogenetic relationships among species, we evaluated each correlation after accounting for phylogeny. The evolutionary correlations were tested with the phylogenetically independent contrast (PIC) analysis by using the ‘ape’ package (Paradis and Schliep 2019) in R v.4.3.0 (R Core Team 2020), combining molecular phylogenetic relationships. All PIC regressions were constrained to pass through the origin to align with the theoretical expectations for interpreting evolutionary trait correlations under the Brownian motion model (Felsenstein 1985). This constraint is necessary because PICs are calculated as standardized differences between phylogenetic nodes, which allowing a nonzero intercept would introduce a statistical artefact, and the origin constrained regression ensures that the slope reflects the evolutionary correlation between traits.

Additionally, a principal component analysis (PCA) was performed using the prcomp function in the ‘vegan’ package to test the relationships among floral and leaf traits (Oksanen et al. 2024). The Pearson correlation was used to evaluate the relationship between the first and the second principal component axes (PC1 and PC2). A PCA from flower and leaf traits was conducted, and flower PC1 vs. leaf PC1 were fitted separately for each of epiphytic and terrestrial *Cymbidium* species. In this way, the relative contribution of each trait to PC1 and PC2 and the preference were estimated. The phylogenetic PCA was also conducted, evaluating whether the phylogenetically structured flower and leaf water morphological and physiological traits detected by the phylogenetic PCA evolved in an associated way (E-Vojtkó et al. 2022). The species loading based on the principal components (PCs) was plotted using the package ‘factoextra’ in R v.4.3.0 (Kassambara and Mundt 2020). Relationships were conducted through ‘ggplot2’ package in R v.4.3.0. A permutational multivariate analysis of variance (PERMANOVA) was used to assess whether species from different life forms occupy distinct positions in their multivariate trait syndromes (number of permuted datasets = 999) with the adonis function in the ‘vegan’ package (Anderson 2001).

## Results

### Differences in flower and leaf traits between epiphytic and terrestrial *Cymbidium* species

All of the floral and leaf traits measured varied significantly across the studied species, and the range of variation differed for each trait (Table 1). The CVs were >70% for *T*_flower_  _70_, *T*_leaf 70_, LMT, and LT, while the coefficients of variation were <30% for FMA, petal vein density (PVD), sepal vein density (SVD), LMA, LLET, and LVD (Table 1). The variation of *T*_leaf 70_ was the highest (102.47%), while the variation of SVD was the lowest (21.84%). There were no significant differences in 13 flower traits between the two life forms (Table 2). The six of the eight leaf traits differed between epiphytic and terrestrial *Cymbidium* species. Specifically, the epiphytic *Cymbidium* species had larger LMA, *T*_leaf 70_, LT, LMT, LUEP, and LLEP, whereas LSD (*P* = .36) and LVD (*P* = .57) did not significantly differ between epiphytic and terrestrial *Cymbidium* species (Table 2).

**Table 1.** Descriptive statistics of morphological and physiological traits from flower and leaf for 19 tested *Cymbidium* species.

Traits	Unit	Functional significance	Mean ± SE	Min	Max	CV (%)
Flower mass per unit area (FMA)	g m^−2^	Water availability	51.78 ± 3.37	31.30	86.90	28.37
Floral longevity (FL)	day	Reproductive success	37.86 ± 2.91	25.63	66.98	33.47
Time required for drying of saturated flowers to 70% RWC (*T*_flower_ _70_)	h	Water loss	7.08 ± 1.20	2.75	21.70	73.89
Petal thickness (PT)	μm	Water availability	365.48 ± 34.85	94.05	679.00	41.56
Petal upper epidermal thickness (PUET)	μm	Water conservation	39.99 ± 4.36	9.74	83.58	47.56
Petal lower epidermal thickness (PLET)	μm	Water conservation	35.15 ± 3.68	8.81	67.12	45.63
Petal mesophyll thickness (PMT)	μm	Water storage	291.10 ± 29.24	75.54	556.56	43.78
Petal vein density (PVD)	mm mm^−2^	Water transport	1.36 ± 0.08	0.80	2.10	25.84
Sepal thickness (ST)	μm	Water availability	358.96 ± 35.19	117.51	663.48	42.73
Sepal upper epidermal thickness (SUET)	μm	Water conservation	35.66 ± 3.72	12.80	68.12	45.44
Sepal lower epidermal thickness (SLET)	μm	Water conservation	30.69 ± 2.75	11.27	53.60	39.00
Sepal mesophyll thickness (SMT)	μm	Water storage	296.22 ± 30.23	93.30	552.25	44.49
Sepal vein density (SVD)	mm mm^−2^	Water transport	1.34 ± 0.06	0.75	1.98	20.69
Leaf mass per unit area (LMA)	g m^−2^	Water availability	119.81 ± 7.45	77.47	208.78	27.10
Time required for drying of saturated leaves to 70% RWC (*T*_leaf 70_)	h	Water loss	182.67 ± 42.94	24.18	722.50	102.47
Leaf mesophyll thickness (LMT)	μm	Water storage	375.88 ± 74.08	129.74	1531.63	85.91
Leaf thickness (LT)	μm	Water availability	416.76 ± 76.49	158.46	1607.70	80.00
Leaf upper epidermal thickness (LUET)	μm	Water conservation	15.65 ± 1.50	8.68	32.25	41.66
Leaf lower epidermal thickness (LLET)	μm	Water conservation	12.57 ± 0.79	7.20	19.02	27.33
Leaf stomatal density (LSD)	no. mm^−2^	Water loss	107.84 ± 7.82	52.35	175.53	31.60
Leaf vein density (LVD)	mm mm^−2^	Water transport	1.40 ± 0.07	0.76	1.93	21.84

CV, coefficient of variation.

**Table 2.** Difference in flower and leaf traits between terrestrial and epiphytic *Cymbidium* species.

Organ	Trait	Epiphytic	Terrestrial	Significance (*P*)
Flower	FMA	54.91 ± 4.99	46.43 ± 2.57	.24
	FL	42.27 ± 4.09	30.30 ± 1.14	.05
	*T*_flower70_	8.50 ± 1.78	4.64 ± 0.44	.12
	PT	352.88 ± 50.03	387.10 ± 43.58	.65
	PUET	40.99 ± 6.08	38.260 ± 6.18	.77
	PLET	35.18 ± 4.96	35.11 ± 5.74	.99
	PMT	276.68 ± 41.08	315.62 ± 38.72	.54
	PVD	1.43 ± 0.12	1.25 ± 0.08	.29
	ST	353.68 ± 48.53	368.01 ± 51.57	.85
	SUET	36.56 ± 4.82	34.13 ± 6.23	.76
	SLET	32.12 ± 3.74	28.24 ± 3.97	.51
	SMT	284.43 ± 41.56	316.43 ± 43.67	.62
	SVD	1.37 ± 0.10	1.28 ± 0.05	.51
Leaf	LMA	131.96 ± 5.98	98.98 ± 3.21	<.001
	*T*_leaf70_	241.33 ± 29.43	86.61 ± 11.09	<.001
	LT	522.77 ± 51.96	286.28 ± 32.29	<.001
	LMT	477.36 ± 50.46	249.30 ± 11.47	<.001
	LUET	18.18 ± 0.98	13.44 ± 0.49	<.001
	LLET	14.00 ± 0.49	11.43 ± 0.37	<.001
	LSD	113.51 ± 8.43	98.11 ± 15.78	.36
	LVD	1.37 ± 0.10	1.46 ± 0.10	.57

FMA, flower mass per unit area; FL, floral longevity; *T*_flower_  _70_, time required for drying of saturated flowers to 70% RWC; PT, petal thickness; PUET, petal upper epidermal thickness; PLET, petal lower epidermal thickness; PMT, petal mesophyll thickness; PVD, petal vein density; ST, sepal thickness; SUET, sepal upper epidermal thickness; SLET, sepal lower epidermal thickness; SMT, sepal mesophyll thickness; SVD, sepal vein density; LMA, leaf mass per unit area; *T*_leaf 70_, time required for drying of saturated leaves to 70% RWC; LMT, leaf mesophyll thickness; LT, leaf thickness; LUET, leaf upper epidermal thickness; LLET, leaf lower epidermal thickness; LSD, leaf stomatal density; LVD, leaf vein density.

### Correlations in floral and leaf traits between epiphytic and terrestrial *Cymbidium* species

**Figure 1.**
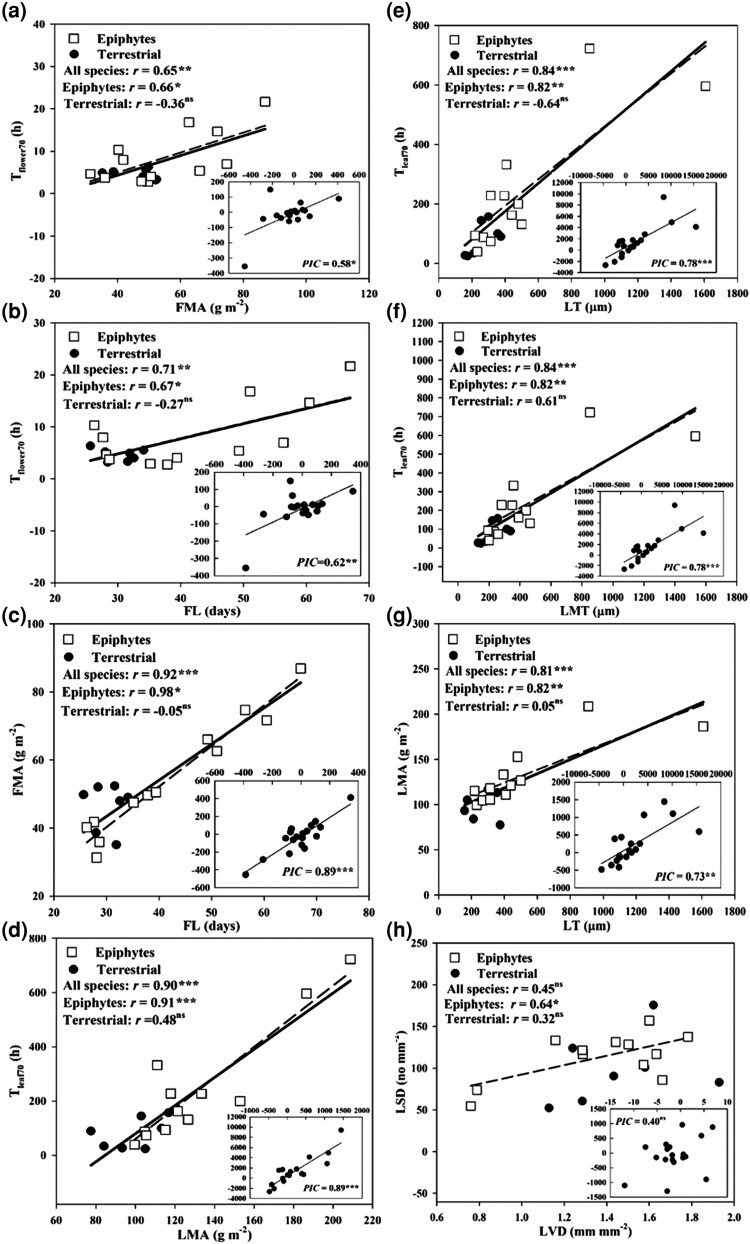
Traditional and PICs correlations between the time required for drying of saturated flowers to 70% RWC (*T*_flower70_) with FMA (a), FL (b), and the relationship of FMA and FL (c) in epiphytic and terrestrial *Cymbidium* species; the correlations between the time required for drying of saturated leaves to 70% RWC (*T*_leaf70_) with LMA (d), LT (e), LMT (f); the relationship between LMA and LT (g); the correlation between LSD and LVD (h). The dashed line represents epiphytic species, the bold solid line represents all species, the squares represent epiphytic species, and the circles represent terrestrial. *P < .05, **P < .01, ***P < .001.

Significant correlations were found among morphological and physiological traits in both flowers and leaves. In flowers, *T*_flower_  _70_ was positively correlated with FMA and FL across all the studied *Cymbidium* species and epiphytic species (Fig. 1a and b), and FL was positively correlated with FMA for all studied species and epiphytic species, but the significant relationship was not found in terrestrial species (Fig. 1c). When the phylogenetic relationship was taken into account, there were significantly positive correlations among FL, FMA, and *T*_flower_  _70_ across all the studied species (Fig. 1, Supplementary Table S2). In leaves, *T*_leaf 70_ was significantly correlated with LMA, LT, and LMT for all tested *Cymbidium* species and epiphytic species but not for terrestrial species (Fig. 1d–f). LMA was correlated positively with LT for all species but not for terrestrial species (Fig. 1g). There were significantly positive correlations among LMA, LT, LMT, and *T*_leaf 70_ by the PICs’ method for all the studied *Cymbidium* species (Fig. 1, Supplementary Table S3). LSD was positively correlated with LVD only in epiphytic species (Fig. 1h). However, the correlation between LSD and LVD was insignificant across all the studied species whether or not phylogeny was considered (Fig. 1, Supplementary Table S3).

### Multivariate analysis of floral and leaf traits

**Figure 2.**
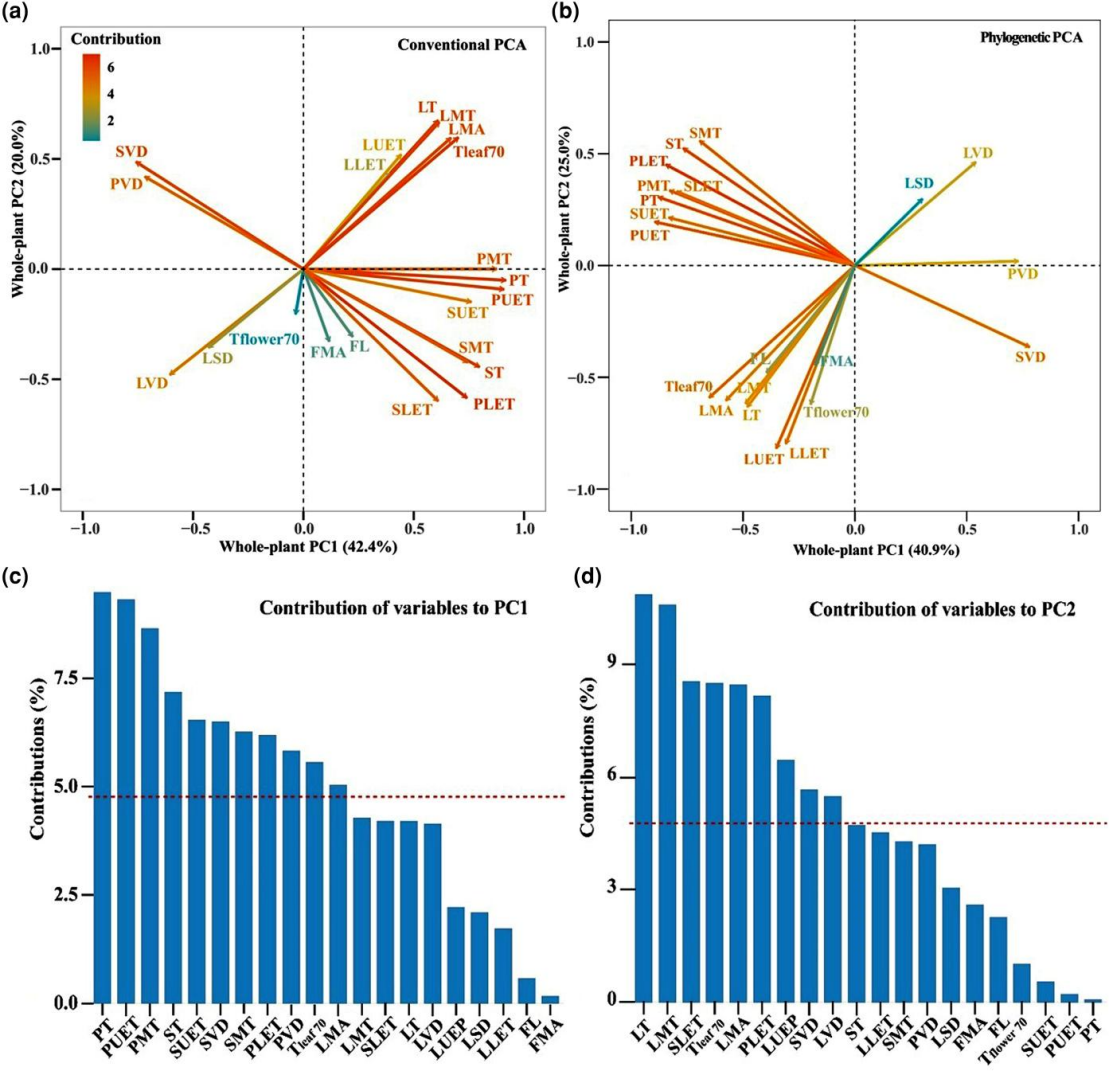
Arrangement of the 13 floral and 8 leaf morphological and physiological traits along the first 2 principal component axes constructed based on (a) *Cymbidium* species traits means and (b) PICs and the contribution of each variable to the first (c) and second (d) PCs based on traditional phylogenetic PCA. The red horizontal dashed lines in (c) and (d) indicate the average contribution of each variable to the corresponding PC, FMA, FL, time required for drying of saturated flowers to 70% RWC (*T*_flower_  _70_), PT, PUET, PLET, PMT, PVD, ST, SUET, SLET, SMT, SVD, LMA, time required for drying of saturated leaves to 70% RWC (*T*_leaf 70_), LMT, LT, LUET, LLET, LSD, and LVD.

**Figure 3.**
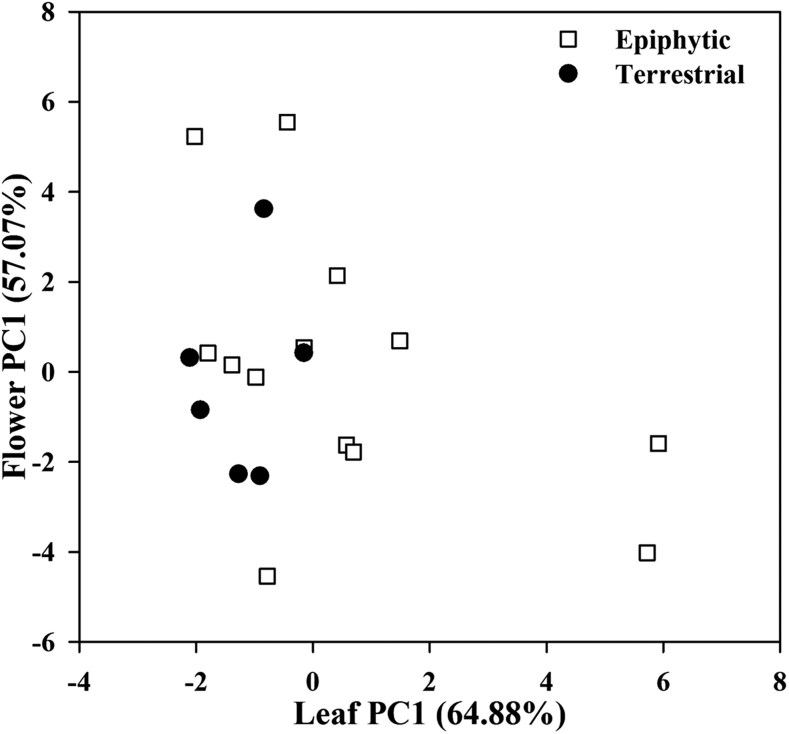
Correlations between flower PC1 and leaf PC1 with respect to epiphytic *Cymbidium* species, terrestrial species, and all species. Pearson correlation coefficients (*r*) were −0.49 (*P* = .11), 0.18 (*P* = .69) and −0.36 (*P* = .13), respectively. The squares represent epiphytic species, and the circles represent terrestrial.

The PCA base on 13 floral traits and 8 leaf traits for the 19 *Cymbidium* species indicated that the first and second component axes explained 42.4% and 20.0% of the total variations, respectively (Fig. 2a). The first PC was mainly explained by floral traits such as PT (9.48%), PUET (9.30%), PMT (8.64%), ST (7.17%), SUET (6.53%), SVD (6.49%), SMT (6.26%), PLET (6.18%), and PVD (5.82%; Fig. 2c). The second PC was mainly contributed by leaf-related traits such as LT (10.86%), LMT (10.58%), SLET (8.55%), *T*_leaf 70_ (8.50%), and LMA (8.45%; Fig. 2d). The patterns of the phylogenetic PCA were generally consistent with those of conventional PCA, except FL, FMA, and *T*_leaf 70_ were orthogonal to leaf traits (Fig. 2b). The result of PERMANOVA indicated that species with different life forms did not show a significant separation (*P* = .42; Supplementary Fig. S2). Additionally, there were no significant correlations between leaf PC1 and flower PC1 for epiphytic *Cymbidium* species (*r* = −0.49, *P* < .11), terrestrial *Cymbidium* species (*r* = 0.18, *P* = .69), and all *Cymbidium* species (*r* = −0.36, *P* = .13; Fig. 3).

## Discussion

### Differences in floral and leaf traits between epiphytic and terrestrial species

Our study focused on *Cymbidium* species with two different life forms facing extreme differences in water and nutrient resource availability (Motomura et al. 2008). Compared with the stable growing environment of terrestrial *Cymbidium* species, epiphytic species usually face drought and lower nutrient availability (Motomura et al. 2008). The leaf trait results of our present study were similar to previous findings (Zhang et al. 2015, Li et al. 2018). Epiphytic orchids exhibited significantly different water-related leaf traits from terrestrial species, with the former exhibiting trait values indicative of greater drought tolerance in this study. The ability to retain water in the leaves plays an important role in maintaining a water balance in epiphytic species (Zhang et al. 2015). Leaf epidermis thickness and LVD significantly increased under water stress in an epiphytic orchid, *Dendrobium chrysanthum* (Qi 2020). Although drought reduces flower size, flower number, and floral reward (Galen et al. 1999, Kuppler and Kotowska 2021), a comparative study on floral morphological and water physiological traits under drought stress by control experiments remains rather scarce.

In this study, we found that there were no differences in floral traits between epiphytic and terrestrial *Cymbidium* species, suggesting that flowers and leaves may be physiologically modular under environmental stress (Roddy et al. 2013), and that floral trait may be relatively unaffected by life form, i.e. the floral trait is remarkably stable. A previous study suggested that flower and fruit water relations are not affected by large variations of plant water status (Trolinder et al. 1993). The lack of difference in floral traits between the two life forms may suggest that the mechanism of flower opening is not related to a water potential gradient or increased solutes (Trolinder et al. 1993). Our result supports the explanation regarding the difference in flower and leaf water-related traits from Roddy et al. (2013). Specifically, these results suggest that vegetative and reproductive organs may develop independently as a consequence of the selective pressures they experienced and the functions they performed.

Leaf morphological and physiological traits have often been investigated to determine how plants adapt to different growing environments (Hao et al. 2010, Fu et al. 2012, Zhang et al. 2015, Guan et al. 2023). The present results show clear differences in leaf traits between epiphytic and terrestrial *Cymbidium* species. Epiphytic species have a higher LMA and the time required for drying of saturated leaves to 70% RWC, as well as thicker leaves and epidermis compared with the terrestrial species. Such leaves can store more water, thereby enabling them to maintain high water potential during drought periods (Stratton et al. 2000, Zotz and Bader 2009, Ogburn and Edwards 2010, Zhang et al. 2015). In this study, we indicated that leaf traits related to the water transport (vein density) and transpiration (stomatal density) did not significantly differ between the epiphytic and the terrestrial *Cymbidium* species. Our finding is not line with previous researches that terrestrial species have greater values for LSD and LVD than epiphytic species (Rada and Jaimez 1992, Zhang et al. 2012, 2015). An earlier report also found that LSDs are higher in nonsucculent species than in succulent species (von Willert et al. 1992).

However, our result is consistent with a previous study that show insignificant differences in LSD and LVD between the two life forms (Zhang et al. 2015). *Cymbidium* species are a perennial herbaceous plant, usually with succulent roots and pseudobulbs and a thin leathery of rigid thick leathery leaves. The possible explanation for this lack of difference in stomatal and vein density between epiphytic and terrestrial *Cymbidium* species is that the high storage capacity of *Cymbidium* species may temporarily help them get through drought periods, and an increased capacity of water storage in leaves, stems, and roots probably plays a more important role in maintaining the whole-plant water balance than the investment in water loss and transport systems (Zhang et al. 2015, Yang et al. 2016, Li and Zhang 2019). Thus, increasing water storage may play an important role in how plants respond to periodic water shortages, helping them to avoid water stress (Ogburn and Edwards 2010, Pivovaroff et al. 2014, Zhang et al. 2015). Life forms influenced the differences in leaf traits in *Cymbidium* species. These results are similar to previous studies that found physiological and morphological divergences between terrestrial and epiphytic species (Hao et al. 2010, Watkins and Cardelús 2012, Zhang et al. 2015).

### Correlations in floral and leaf traits

The time required for drying of saturated leaves to 70% RWC is a threshold for physiological damage, which is related to the resistance of the cuticle to water loss (Hao et al. 2010). Previous studies have shown that the time required for drying of saturated leaves to 70% RWC of hemiepiphytic *Ficus* species is significantly longer than that of nonhemiepiphytic *Ficus* species (Holbrook and Putz 1996, Hao et al. 2010). Leaves of epiphytic species have a higher LMA than those of terrestrial species. Such leaves have thicker spongy parenchyma that can store more water, allowing them to maintain high water potential under water stress (Stratton et al. 2000, Zotz and Bader 2009, Ogburn and Edwards 2010). Our results showed that the time required for drying of saturated flowers to 70% RWC was correlated with FMA and FL, but only in epiphytic species. This relationship suggests that epiphytic species may mitigate flower water loss by increasing the retention time of water in flowers when faced with limited carbon and water resources. The time required for drying of saturated flowers to 70% RWC represents the water retention time in flower tissues, and maintaining high FMA and long FL requires abundant carbon and water investments throughout the flower lifespan. This implies that flowers of epiphytic species rely on their own internal stored water to maintain floral water balance, making epiphytic species more physiologically decoupled from the environment than terrestrial species.

In our study, the time required for drying of saturated leaves to 70% RWC was significantly associated with LT and LMT only in epiphytic species, possibly because water is stored as a cushion to resist the transpiration stream. Because of their greater water storage capacity, epiphytic orchids can alleviate stress associated with periodic low water availability (Zhang et al. 2015). Our results showed that LSD was positively correlated with LVD in epiphytic species. Higher LVD enhances water transport and allows higher leaf conductances to CO_2_ and water (Brodribb and Jordan 2011); at the same time, higher stomatal density increases gas exchange and thus enhances leaf photosynthesis under water availability conditions, and the coordinated development of veins and stomata may make them to adapt epiphytic habitats. In contrast, terrestrial species did not show a strong relationship between LSD and LVD. The terrestrial orchids are able to absorb water from the soil (Liu et al. 2006), and they may not need to rely exclusively on rapidly transport water by leaf vein to maintain water balance and gas exchange. In addition, the terrestrial species may employ other adaptive mechanisms to cope with drought, such as enhanced antioxidant activity and increased concentrations of abscisic acid and jasmonates (Li et al. 2018). The inconsistent trait correlation between epiphytic and terrestrial species might indicate different adaption mechanisms (Zhang et al. 2015). The tight association between stomatal and vein density suggests that maintaining dynamic water balance is more critical for epiphytic species to adapt water-limited conditions. Unlike the survival strategy of terrestrial species, epiphytic species prefer to keep leaf water balance between requirement and supply, thus providing a stable growing environment by employing a conservative water use strategy. The relationship between LSD and LVD might be altered according to plant demands.

### Multivariate analysis of floral and leaf traits

The coupling associations among root, leaf, stem, and wood functional characteristics have been found in some plant species (Zhao et al. 2017, Li et al. 2022). Similarly, the hypothesized root economics spectrum showed that above- and below-ground resource strategies are coordinated (Reich 2014). However, it was worth noting that this decoupling relationship between roots and leaves was observed (Fortunel et al. 2012, Isaac et al. 2017). It can be concluded that there is no consistent evidence of the root economics spectrum mirroring the leaf economics spectrum (Weemstra et al. 2016). In this study, floral traits were not correlated with leaf traits, indicating a decoupling relationship between flower and leaf traits (Roddy et al. 2013, Zhang et al. 2017). This decoupling relationship may reflect different functions of leaves and flowers, supporting the idea of functional modularity between vegetative and reproductive organs (Roddy et al. 2013). Although the two life forms differ in their natural environments, *Cymbidium* species do not diverge from along the PC1 axis. This result supports that the difference in growth environment did not alter the trade-off between plant functional traits in *Cymbidium*.

A recent study showed that the functional traits of ferns are not affected by the environment (Li et al. 2022). However, Zhao et al. (2017) showed that wood and leaf traits of trees and shrubs are influenced by leaf habits and life forms in subtropical species (Chen et al. 2020, Li et al. 2022). It should be noted that flowers are reproductive organs, and their functions differ from those of vegetative organs, such as leaf, woody, and bark. In addition, *Cymbidium* species are herbaceous plants, which are different from woody and shrub plants, and thus do not produce secondary tissues such as wood and bark structures. *Cymbidium* species have usually leathery, fleshy leaves, and succulent roots and stems, and these organs play a role in water storage and retention to maintain water balance. Both LVD and LSD are lower in *Cymbidium* species (Zhang et al. 2015), so the photosynthetic efficiency and water transport capacity are relatively low. On the other hand, woody or shrub plants have a well-developed root system that obtain water from the different deep soils and transport it to the above-ground organs of the plant. The higher LVD and LSD of woody plants allows them to achieve higher photosynthetic rates and water transport efficiencies (Brodribb and Jordan 2011).

## Conclusion

In this study, we found significant differences in leaf morphological and physiological traits between epiphytic and terrestrial *Cymbidium* species. Epiphytic *Cymbidium* species exhibited a higher LMA, greater LT, and greater leaf epidermal and mesophyll thicknesses. These traits suggest an adaptation strategy of water conservation and storage. Evidently, in the life history of epiphytes, leaf traits play a vital role in enabling them to adapt water-limited habitats. Conversely, terrestrial species, which have more stable access to soil-sourced water, do not display such extreme values for these traits. Notably, despite the differences in leaf traits, floral traits did not vary significantly between epiphytic and terrestrial *Cymbidium* species. This implies that flower development may be less directly influenced by the immediate water-related factors. It is possible that in the life histories of both epiphytic and terrestrial species, floral traits are more conserved and perhaps determined by other factors such as pollination strategies, genetic constraints, or long-term evolution. The decoupling of floral and leaf traits further supports the idea that different organs of plants have distinct developmental and adaptive strategies.

## Supplementary Material

plaf033_Supplementary_Data

## Data Availability

The data of this article are available at https://osf.io/56hce/ and in the Supplementary data.
