# Supplementary material for: The water-related traits of flowers are more conservative than those of leaves for epiphytic and terrestrial species in Cymbidium, Orchidaceae
Source: AoB Plants. 2025 Jun 24;17(4):plaf033. doi: 10.1093/aobpla/plaf033 (PMC12268501; doi:10.1093/aobpla/plaf033)
Supplement: plaf033_Supplementary_Data [file plaf033_supplementary_data.pdf]

Supporting information

Table S1. Life forms, abbreviation, and native habitat features of the tested *Cymbidium* species in the present study.

Species	Code	Section	Elevation (m)	Endemic habitat
Epiphytic				
C. erythraeum	1	*Iridorchis*	1400–2800	Trees or rocks on forest margins or in forests
C. sichuanicum	2	*Iridorchis*	1200–1600	Trees in forests or rocks on forest edges
C. mastersii	3	*Eburnea*	1600–1800	Trees or rocks in forests
C. dayanum	4	*Himantophyllum*	300–1600	Trees in open forests, cliffs along stream sides
C. aloifolum	5	*Cymbidium*	100–1100	Large branches or tree trunks in open forests or thickets, or cliffs along stream sides of valleys
C. tracyanum	6	*Iridorchis*	1200–1900	Tree trunks in forests, rocks by stream sides
C. maguanense	7	*Eburnea*	1000–1800	Trees in forests
C. bicolor	8	*Cymbidium*	100–1600	Trees in forests and thickets
C. cochleare	9	*Cyperorchis*	300–1800	Trees in forests
C. changningense	10	*Eburnea*	1700	Trees on forest margins or shaded rocks
C. devonianum	11	*Bigibbarium*	1500	Rocks in open forests
C. elegans	12	*Cyperorchis*	1700–2800	Trees in forests, cliffs
Terrestrial				
C. goeringii	13	*Jensoa*	300–2200	Rocky slopes, forest margins
C. ensifolium	14	*Jensoa*	600–1800	Open forests, thickets, grassy places along valleys
C. sinense	15	*Jensoa*	300–2000	Forests and well-drained shaded places in thickets
C. teretipetiolatum	16	*Nanula*	1000	Open forests
C. insigne	17	*Iridorchis*	1700–1900	Grassy and rocky places in open forests
C. cyperifolium	18	*Axillaria*	700–1800	Forests
C. lancifolum	19	*Geocymbidium*	300–2000	Open forests, bamboo forests, broad-leaved forests, forest margins

Table S2. Correlations of Pearson's correlation (upper right of the diagonal) and phylogenetic independent contrast correlations (lower left of the diagonal) among floral traits from 19 *Cymbidium* species.

	FMA	FL	$T_{flower70}$	PT	PUET	PLET	PMT	PVD	ST	SUET	SLET	SMT	SVD
FMA		0.92***	0.65**	-0.004	-0.04	0.18	-0.05	-0.24	0.23	0.03	0.23	0.24	-0.15
FL	0.86***		0.71**	0.004	0.13	0.35	-0.07	-0.29	0.23	0.20	0.44	0.19	-0.22
$T_{flower70}$	0.58*	0.62**		-0.24	-0.14	0.10	-0.29	-0.20	-0.04	-0.17	0.27	-0.08	-0.02
PT	-0.05	0.06	-0.18		0.84***	0.64**	0.99***	-0.71**	0.79***	0.65**	0.47*	0.77**	-0.77***
PUET	-0.08	0.16	-0.18	0.89***		0.80***	0.78***	-0.60**	0.72***	0.91***	0.64**	0.64**	-0.700**
PLET	-0.03	0.21	-0.003	0.80***	0.82***		0.55*	-0.72**	0.81***	0.79***	0.92***	0.73***	-0.78***
PMT	-0.06	0.01	-0.24	0.99***	0.86***	0.74***		-0.68**	0.75***	0.57*	0.38	0.74***	-0.74***
PVD	-0.29	-0.44	-0.54*	-0.52*	-0.51*	-0.69**	-0.48		-0.65**	-0.41	-0.69**	-0.62**	0.89***
ST	0.08	0.16	-0.04	0.81***	0.70**	0.89***	0.79***	-0.54*		0.63**	0.65**	0.98***	-0.79***
SUET	-0.09	0.15	-0.23	0.83***	0.96***	0.80***	0.78***	-0.38	0.67**		0.62**	0.54*	-0.49*
SLET	-0.06	0.19	0.20	0.71**	0.68**	0.93***	0.63**	-0.76***	0.78***	0.64**		0.54*	-0.72**
SMT	0.11	0.16	-0.05	0.74***	0.62**	0.85***	0.73**	-0.49*	0.99***	0.59**	0.72**		-0.76***
SVD	-0.20	-0.36	-0.13	-0.70**	-0.71**	-0.86***	-0.68**	0.81***	-0.81***	-0.60**	-0.80***	-0.77***	

Note: flower mass per unit area (FMA), floral longevity (FL), time required for drying of saturated flowers to 70% RWC ($T_{flower\ 70}$), petal thickness (PT), petal upper epidermal thickness (PUET), petal lower epidermal thickness (PLET), petal mesophyll thickness (PMT), petal vein density (PVD), sepal thickness (ST), sepal upper epidermal thickness (SUET), sepal lower epidermal thickness (SLET), sepal mesophyll thickness (SMT), sepal vein density (SVD). * $P < 0.05$, ** $P < 0.01$, *** $P < 0.001$.

Table S3. Correlations of Pearson's correlation (upper right of the diagonal) and phylogenetic independent contrast correlations (lower left of the diagonal) among leaf traits from 19 *Cymbidium* species.

	LMA	T_{leaf70}	LT	LMT	LUET	LLET	LSD	LVD
LMA		0.90***	0.81***	0.81***	0.65**	0.59**	-0.48*	-0.57*
T_{leaf70}	0.89***		0.84***	0.84***	0.65**	0.60**	-0.38	-0.63**
LT	0.73**	0.78***		0.99***	0.51*	0.43	-0.41	-0.65**
LMT	0.73**	0.78***	0.99***		0.50*	0.42	-0.41	-0.64**
LUET	0.60**	0.70**	0.51*	0.50*		0.89***	-0.17	-0.51*
LLET	0.63**	0.63**	0.45	0.44	0.92***		-0.08	-0.37
LSD	-0.42	-0.22	-0.21	-0.21	-0.28	-0.25		0.45
LVD	-0.54*	-0.61**	-0.64**	-0.63**	-0.60**	-0.46	0.40	

Note: leaf mass per unit area (LMA), time required for drying of saturated leaves to 70% RWC (T_{leaf70}), leaf thickness (LT), leaf mesophyll thickness (LMT), leaf upper epidermal thickness (LUET), leaf lower epidermal thickness (LLET), leaf stomatal density (LSD), leaf vein density (LVD). * P < 0.05, ** P < 0.01, *** P < 0.001.

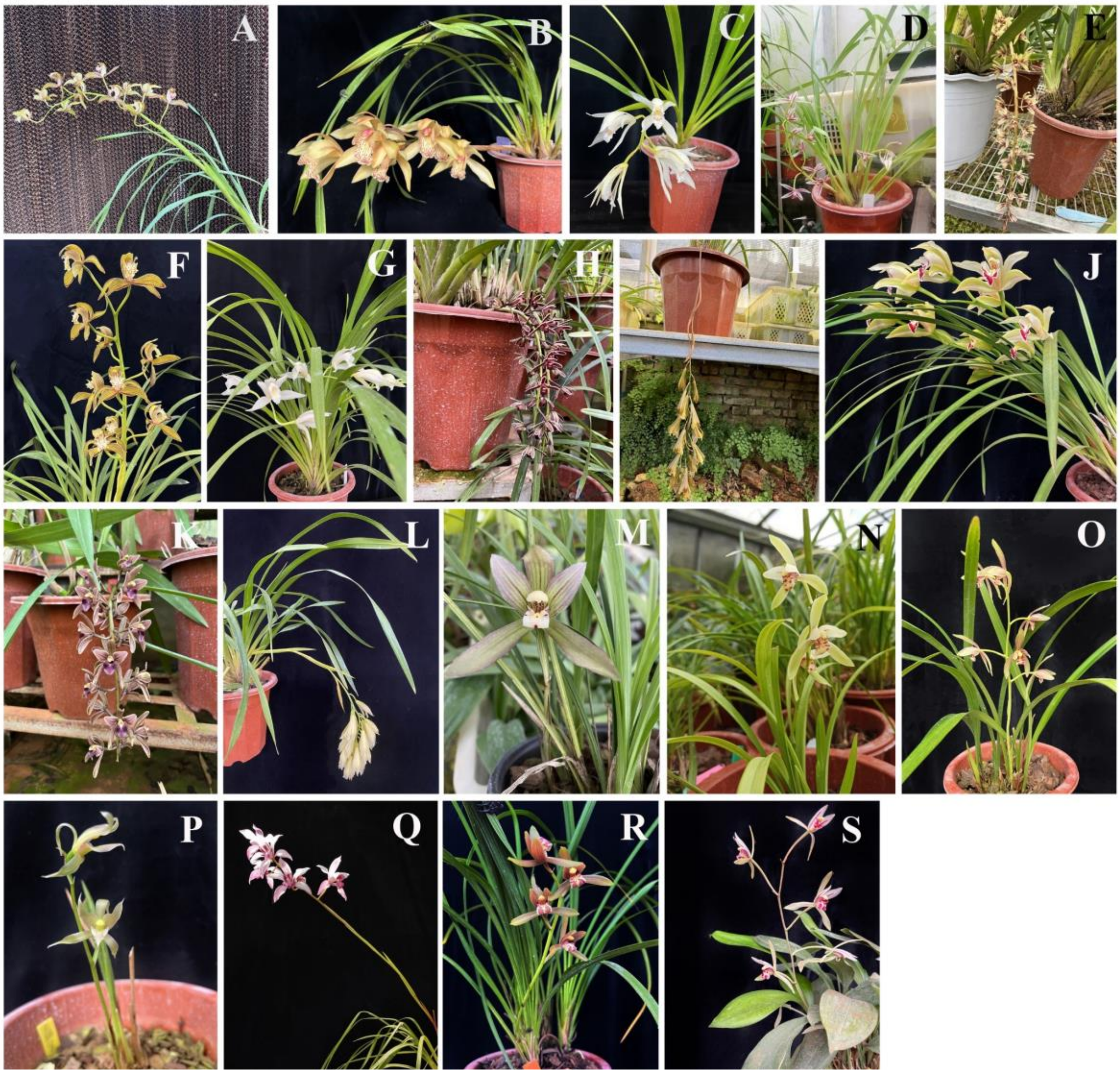

Figure S1. The 19 *Cymbidium* species investigated in this study. (A) *C. erythraeum*; (B) *C. sichuanicum*; (C) *C. mastersii*; (D) *C. dayanum*; (E) *C. aloifolum*; (F) *C. tracyanum*; (G) *C. maguanense*; (H) *C. bicolor*; (I) *C. cochleare*; (J) *C. changningense*; (K) *C. devonianum*; (L) *C. elegans*; (M) *C. goeringii*; (N) *C. ensifolium*; (O) *C. sinense*; (P) *C. teretipetiolatum*; (Q) *C. insigne*; (R) *C. cyperifolium*; (S) *C. lancifolum*

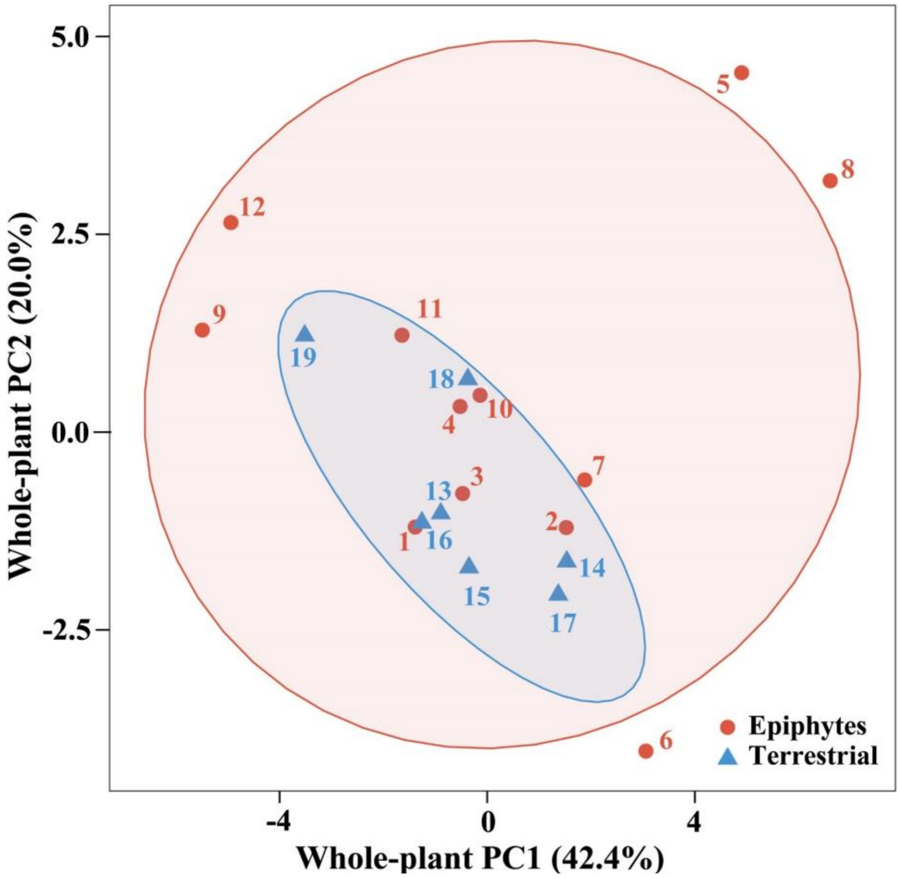

Figure S2. The factor loading of each species based on flower and leaf traits along PC1 and PC2 for epiphytic species (red circles) and terrestrial species (blue triangles), circles represent epiphytic species, and triangles represent terrestrial.
